# Evaluation of ChatGPT-Generated Simplifications of Shoulder MRI Reports: Assessments by Orthopedic Surgeons and Non-Healthcare Volunteers

**DOI:** 10.3390/diagnostics16213609

**Published:** 2026-11-01

**Authors:** Turgut Emre Erdem, Alper Kirilmaz, Furkan Dogan, Fatih Tahak, Muhammed Furkan Kucuksen, Muhammed Yıldız, Selcuk Yilmaz, Dilek Yapar, Aliekber Yapar

**Affiliations:** 1 Department of Orthopedics and Traumatology, Faculty of Medicine, Akdeniz University, Antalya 07070, Türkiye; furkandogan@gmail.com (F.D.); selcukyilmaz@akdeniz.edu.tr (S.Y.); aliekberyapar@yahoo.com (A.Y.); 2 Department of Orthopedics and Traumatology, Konya City Hospital, Konya 42020, Türkiye; alprkrlmz@gmail.com (A.K.); f.kucuksen2013@hotmail.com (M.F.K.); 3 Department of Orthopedics and Traumatology, Karapinar State Hospital, Konya 42400, Türkiye; dr.fth.th@gmail.com; 4 Department of Orthopedics and Traumatology, Necip Fazıl City Hospital, Turkish Ministry of Health, Kahramanmaraş 46050, Türkiye; muhammed.yildiz82@gmail.com; 5 Department of Medical Informatics, Institute of Health Sciences, Akdeniz University, Antalya 07070, Türkiye; dilekyapar@hotmail.com

**Keywords:** artificial intelligence, ChatGPT, large language model, patient-friendly communication, radiology report, shoulder MRI

## Abstract

**Background/Objectives:** To evaluate ChatGPT-generated (GPT-5) simplifications of shoulder MRI reports from the perspectives of orthopedic surgeons and non-healthcare volunteers. **Methods**: In this single-center retrospective study, 100 de-identified shoulder MRI reports issued between June 2024 and June 2025 were simplified with ChatGPT in February 2026 using a standardized prompt. Between 10 March and 10 April 2026, three orthopedic surgeons independently rated each simplified text against the original report for accuracy, completeness, understandability, and misleading potential on a 5-point Likert scale (higher scores indicating better ratings). In a second arm, 100 adult non-healthcare volunteers each reviewed one simplified report and completed a six-item questionnaire on perceived clarity, comprehensibility, usefulness, and appropriateness. **Results**: Median expert scores were 5.0 in all domains, and all three raters assigned scores ≥ 4 in 86–98% of reports. Gwet’s AC2 ranged from 0.93 to 0.98, whereas Krippendorff’s α ranged from −0.02 to 0.05. Among volunteers, agreement ranged from 47% for understanding report terms to 80% for the appropriateness of the text. Health literacy correlated positively with most items (r = 0.329–0.429, *p* ≤ 0.001), and age correlated negatively with understanding report terms (r = −0.352, *p* < 0.001). **Conclusions**: The simplified reports were rated favorably by orthopedic surgeons and were perceived as useful by non-healthcare volunteers. However, the study was limited by its single-center design, the use of a single model and prompt, the absence of a comparison with the original reports in the volunteer arm, and the reliance on self-reported rather than objectively tested comprehension; therefore, it does not demonstrate improved patient comprehension or clinical safety. Such texts should supplement, not replace, the original report and physician consultation.

## 1. Introduction

Increased access to electronic health records and patient portals has made it easier for patients to directly view their imaging reports; however, this does not necessarily mean that such reports are understandable at the patient level. Poor comprehensibility of radiology reports may lead to confusion, increased anxiety, and a greater need for additional explanation in patient–physician communication [1,2]. The internet has become a major source of health information for the public: in a nationally representative U.S. survey, 72% of internet users reported having looked online for health information within the previous year, and 35% of adults had gone online specifically to try to figure out a medical condition [3]. During the COVID-19 pandemic, when diagnostic and treatment protocols were uncertain, many healthcare professionals and members of the public turned to the internet, and to YouTube in particular, for health-related information [4,5]. More recently, 17% of U.S. adults reported using AI chatbots at least once a month to find health information and advice, although only about one-third were confident that this information was accurate [6]. Because the quality of online and AI-generated health information shapes how individuals perceive their condition and make health-related decisions, inaccurate or misleading content may lead to consequences that are difficult to reverse.

In recent years, large language models (LLMs) and generative artificial intelligence systems have shown considerable potential for rephrasing medical texts into simpler, more readable, and more patient-friendly language [7,8]. Berzolla et al. reported that AI-based simplification of MRI reports significantly improved patient comprehension and that these simplified reports were preferred for clarity, although perceived trust was lower compared with conventional reports [9].

The broader radiology literature beyond musculoskeletal imaging has demonstrated a similar trend. Van Driel et al. showed that radiology reports simplified with GPT-4 could improve patient comprehension and were feasible in terms of patient appropriateness [10]. Sunshine et al. found that radiology report summaries generated by ChatGPT were promising in terms of technical accuracy, patient satisfaction, and readability; however, some summaries carried risks of semantic drift, oversimplification, or lack of contextual nuance [11]. In another study of oncology patients, simplified radiology reports improved patients’ understanding of their disease and their confidence in the report itself [12]. Despite this growing literature, studies specifically evaluating the simplification of shoulder MRI reports from both expert and layperson perspectives remain limited.

Shoulder MRI reports contain numerous unfamiliar and complex terms for patients, including rotator cuff pathologies, labral lesions, tendinosis, partial- or full-thickness tears, bursitis, and degenerative changes; therefore, the safe and accurate translation of these reports into patient-friendly language is of particular importance. Moreover, improving readability alone is not sufficient: simplified outputs must also be evaluated for accuracy, completeness, understandability, and potential to mislead [11,13]. Thus, AI-generated simplified MRI reports should be evaluated from both expert and end-user perspectives to determine their suitability for clinical use [9,11,12]. Because musculoskeletal complaints such as shoulder pain are common in the general population, digital health information on these conditions should be accessible to readers with a wide range of ages, educational backgrounds, and health literacy levels.

ChatGPT was selected for this study because it is one of the most widely known and used publicly available LLM-based chatbots; in a 2025 nationally representative survey, 34% of U.S. adults reported having used ChatGPT, approximately double the proportion in 2023 [14]. It has also been the model most frequently evaluated in previous studies of radiology report simplification [2,11], which facilitates comparison with the existing literature. Other widely used platforms, such as Gemini, Microsoft Copilot, Perplexity, and Grok, may perform differently [7,8]; a comparison between models was beyond the scope of this study.

The primary objective of this study was to evaluate ChatGPT-generated simplifications of shoulder MRI reports in terms of accuracy, completeness, understandability, and misleading potential, as assessed by orthopedic specialists, and to evaluate the perceived comprehensibility and usefulness of these simplified reports among non-healthcare volunteers. We hypothesized that ChatGPT could generate simplified shoulder MRI reports that are clinically acceptable to specialists while remaining useful and appropriate for lay readers, thereby addressing a specific gap in the literature on patient-centered communication in musculoskeletal radiology.

## 2. Materials and Methods

### 2.1. Study Design and Ethical Approval

This study was designed as a single-center, retrospective observational study to evaluate the performance of an LLM in simplifying shoulder magnetic resonance imaging (MRI) reports into patient-friendly language. The study protocol was approved by the Institutional Review Board of Akdeniz University Faculty of Medicine (approval code: TBAEK-37, date of approval: 29 January 2026). Because of the retrospective design and the use of fully de-identified radiology reports, the requirement for informed consent from the patients whose reports were used was waived by the ethics committee. Written informed consent was obtained from all volunteers before participation. The simplified reports were generated between 15 and 28 February 2026, and the expert and volunteer evaluations were conducted between 10 March and 10 April 2026. The study was conducted in accordance with the principles of the Declaration of Helsinki.

### 2.2. Selection of Shoulder MRI Reports

Shoulder MRI reports issued between June 2024 and June 2025 were retrospectively retrieved from the institutional radiology archive. Reports were selected consecutively from the archive database, including adult patients (≥18 years) who underwent non-contrast shoulder MRI. Post-operative reports, pediatric reports, and reports with incomplete documentation were excluded. Of the 132 consecutive reports screened, 32 were excluded for these reasons, and 100 reports were included in the study (Figure S1). The original reports were generated during routine clinical care at a tertiary university hospital, where MRI reports are routinely prepared by a radiologist and reviewed and approved by a senior academic radiologist. These existing clinical reports were not re-reported for research purposes. All examinations were performed on a 1.5-T MRI scanner (MAGNETOM Avanto, Siemens Healthcare, Erlangen, Germany). The patients whose reports were included were aged 18–92 years; 54% were female and 46% were male. The reports covered findings commonly encountered in routine shoulder MRI practice, including rotator cuff tears, tendinosis, and labral lesions; however, the frequency of individual findings was not systematically extracted.

Before inclusion in the study, all reports were fully de-identified by removing names, identification numbers, dates, and any information that could reveal patient identity. The original radiologist-authored report texts were preserved and used as the reference standard for all subsequent evaluations. Because the aim of the study was to evaluate the fidelity of the simplified text to its source rather than diagnostic accuracy, the original report served as the reference standard for content; the MRI images were not re-interpreted, and the original reports were not independently verified against the images.

### 2.3. Generation of Simplified Reports

Each original shoulder MRI report was entered individually into ChatGPT (GPT-5; OpenAI, San Francisco, CA, USA), accessed between 15 February 2026 and 28 February 2026 via the web interface using default model settings (generation parameters such as temperature are not user-adjustable in this interface). To prevent potential context carry-over, each report was processed in a new, independent conversation session. A standardized prompt, predetermined by the research team, was used for all reports:

‘‘A shoulder MR report of a patient with shoulder pain is presented. Summarize this report in a way that can be understood by a person with a high school education level. If possible, organize the explanation under the following headings: (1) What does this MR result generally show? (2) What are the normal and abnormal findings presented in the report? (3) Do all abnormal findings require treatment? (4) What is generally recommended for treatment? (5) What should the patient do and avoid during this process?’’

The purpose of this prompt was to convert the technical content of the original radiology report into accessible, patient-friendly language while preserving its core clinical meaning. A structured and relatively detailed prompt was preferred to ensure standardization of model outputs, reduce inter-report variability, and support sufficient comprehensiveness and safety in explanations intended for patient use. However, headings 3–5 of the prompt requested general information on treatment and on what the patient should do or avoid; these items extend beyond linguistic simplification, because such guidance is not usually contained in the radiology report itself. This content was therefore included in the expert assessment, in which the raters judged each output as a whole, including any treatment-related statements, against the original report. These headings were included deliberately because questions about whether treatment is needed and what to do next are among those that patients most often ask after reading an imaging report, and are likely to be posed to AI chatbots; the study therefore aimed to describe how the model responds to such questions. Because the appropriateness of individualized treatment recommendations cannot be judged without clinical information such as symptoms, physical examination findings, and functional demands, which were not available to the model or the raters, this content was not evaluated against a separate clinical reference standard. The AI-generated simplified text of each report was recorded verbatim for analysis, without any manual editing.

### 2.4. Expert Evaluation

The expert evaluation arm consisted of three senior orthopedic surgeons with experience in interpreting shoulder MRI findings in daily clinical practice. Orthopedic surgeons were chosen because they routinely interpret shoulder MRI reports and discuss their implications with patients; musculoskeletal radiologists were not included in the expert panel (see Section 4.1). Each rater independently evaluated every AI-generated simplified report by comparing it with the corresponding original radiology report. Reports were presented to each rater in a randomized order, and raters were blinded to one another’s ratings. The evaluation was performed across four predefined domains:

1. Accuracy: the extent to which the simplified text correctly reflected the findings of the original MRI report.
2. Completeness: the extent to which the important findings of the original report were retained in the simplified text.
3. Understandability: the degree to which the simplified text was clearly written and could be understood by individuals without a healthcare background.
4. Misleading potential: the likelihood that the text could mislead the patient, create unnecessary anxiety, or result in clinically inappropriate interpretations, reflecting its potential for harm. Operationally, raters considered content that could lead to an incorrect understanding of the findings, including overstatement or understatement of severity, false reassurance, unwarranted alarm, or treatment-related statements not supported by the original report. This domain was reverse-scored (see below).

All four domains were evaluated using a 5-point Likert scale. For accuracy, completeness, and understandability, scores ranged from 1 (very poor) to 5 (excellent), with higher scores indicating better performance. For misleading potential, scores ranged from 1 (very high risk) to 5 (very low risk), with lower scores indicating a greater likelihood of misleading the patient; thus, for all four domains, higher scores indicate a more favorable rating. For interpretability, scores ≥ 4 were considered indicative of clinically acceptable performance across all four domains [8,11].

### 2.5. Volunteer Evaluation

The non-expert evaluation arm consisted of 100 adult volunteers without a professional background in healthcare. They were not patients evaluating their own MRI reports; therefore, this arm reflects layperson perceptions of the simplified texts rather than the experience of patients. Each volunteer was presented with one randomly assigned AI-simplified shoulder MRI report and was then asked to complete a structured evaluation form. Restricting each participant to a single report was intended to minimize responder fatigue and reduce potential learning effects. This assessment focused on key domains including clarity, comprehensibility, perceived usefulness, and suitability of the text for patient use. A brief, study-specific questionnaire consisting of six items was developed to evaluate these domains among volunteers. The items were generated based on the study objectives and relevant literature on patient understanding of medical information, covering comprehension of report terminology, identification of important findings, understanding of one’s clinical condition, perceived informativeness, guidance for next steps, and overall appropriateness for patients. The content of the items was reviewed by the research team to ensure clarity and face validity. Responses were collected using a 5-point Likert scale ranging from 1 (strongly disagree) to 5 (strongly agree). For descriptive analyses, responses of 4 and 5 were considered to indicate a positive evaluation. Internal consistency of the questionnaire was assessed using Cronbach’s alpha. Each item was analyzed separately, and no total or composite score was calculated. Because all six items referred to the volunteer’s overall perception of the same simplified text, Cronbach’s alpha was calculated as a supplementary description of the consistency of responses across these related items, rather than as evidence of validity or unidimensionality; given the heterogeneity of the items, it was interpreted only descriptively. Beyond this review, no formal psychometric validation was performed. The form assessed perceived understanding and acceptability rather than objective comprehension.

In addition, basic demographic data were collected from all participants, including age, sex, educational status, previous history of shoulder MRI or shoulder surgery, self-reported difficulty in understanding radiology reports, and self-reported health literacy (perceived ease of understanding health-related information, rated from “very difficult” to “very easy”). These variables were recorded to characterize the study population and to explore the possible influence of individual characteristics on perceived comprehensibility.

### 2.6. Outcome Measures

The primary outcome was the expert-rated quality of the AI-generated simplified reports, assessed across four domains: accuracy, completeness, understandability, and misleading potential. Secondary outcomes were the volunteer-reported (perceived) clarity, comprehensibility, perceived usefulness, and appropriateness of the simplified reports for patient use, as well as the association between volunteer characteristics (age, education, health literacy) and their questionnaire responses.

### 2.7. Statistical Analysis

Statistical analyses were performed using IBM SPSS Statistics for Windows, version 23.0 (IBM Corp., Armonk, NY, USA). Continuous variables were presented as mean ± standard deviation or median (minimum–maximum), depending on distribution characteristics, whereas categorical variables were expressed as number and percentage. Likert-scale data were treated as ordinal: they were summarized as medians (minimum–maximum) and frequencies and analyzed with nonparametric methods; means ± standard deviations are additionally presented for descriptive purposes only. Proportions are reported with 95% confidence intervals (CIs) calculated using the Wilson score method.

The distribution of continuous variables was assessed visually (histograms and probability plots) and with the Kolmogorov–Smirnov and Shapiro–Wilk tests. Because the questionnaire and expert ratings were ordinal, all inferential analyses were performed with nonparametric tests. Inter-rater agreement among the experts was evaluated using exact pairwise agreement, the high score agreement metric, and two chance-corrected reliability coefficients for ordinal data: Krippendorff’s α with ordinal distance [15] and Gwet’s AC2 with quadratic weights [16]. Gwet’s AC2 was included because kappa-type coefficients, including Krippendorff’s α, can be paradoxically low when ratings are concentrated in one or two categories despite high observed agreement [16,17]. The 95% CIs for both coefficients were estimated by nonparametric bootstrapping (5000 resamples of reports), and both coefficients were calculated in Python (version 3.13) using the krippendorff (version 0.9.0) and irrCAC (version 0.4.4) packages (with all five possible rating categories, 1–5, specified), with bootstrap resampling performed in NumPy (version 2.5.3). As a sensitivity analysis, Krippendorff’s α was additionally calculated with nominal and interval distance metrics, Gwet’s coefficient was calculated without weighting (AC1) and with linear weights, and Fleiss’ κ was calculated; all coefficients are reported with bootstrap 95% CIs (Table S1). High score agreement was defined as cases in which all three raters independently assigned scores of ≥4. This approach was preferred due to the ordinal nature of the Likert scale and the observed clustering of ratings in higher categories. Associations of questionnaire item scores with age and self-reported health literacy were assessed using Spearman’s correlation; item scores were compared between educational groups and according to prior shoulder MRI or surgery using the Mann–Whitney U test, and differences according to self-reported difficulty in understanding radiology reports were assessed using the chi-square test. These volunteer subgroup analyses were exploratory; to account for multiple testing, *p*-values were adjusted using the Holm method within each set of six item-level comparisons for a given participant characteristic [18]. A *p*-value < 0.05 was considered statistically significant.

Heatmaps were generated in Python using Matplotlib (version 3.10.8). All Likert categories (1–5), including zero-frequency cells, were retained. Only exact agreement cells (diagonal elements) were color-coded according to agreement percentages, whereas non-agreement cells were displayed with a uniform background.

Given the ordinal nature of the Likert scale and the expected clustering of ratings in higher categories, reliance solely on exact agreement may lead to an underestimation of true inter-rater consistency. Therefore, a high score agreement metric, defined as cases in which all three raters independently assigned scores of 4 or higher (≥4), was additionally calculated as a descriptive measure of joint acceptability (i.e., the proportion of reports rated as acceptable by all three raters); it is not a reliability coefficient. This approach allows for minor variations between adjacent categories while preserving interpretability and has been used to better capture agreement in contexts where distinctions between high ratings are less clinically meaningful.

To summarize expert ratings at the report level, the median of the three ratings was calculated for each domain and used as the consensus score. The median was preferred because the data were based on an ordinal Likert scale and small differences between adjacent scores (e.g., 4 vs. 5) were not considered clinically meaningful.

To characterize errors, all reports that received a rating of ≤3 from at least one rater in any domain were identified and re-examined against the original report, and the type of deviation was characterized (e.g., omission of findings, factual distortion, loss of uncertainty, severity distortion, or inappropriate advice). The low-rated reports are listed with the corresponding domain, score, and rater in Table S2.

## 3. Results

### 3.1. Expert Evaluation

A total of 100 shoulder MRI reports were evaluated by three orthopedic surgeons. Across all four domains, the median score was 5.0 for each rater. Mean scores ranged from 4.6 to 5.0 for accuracy, 4.5 to 4.9 for completeness, 4.9 to 5.0 for understandability, and 4.6 to 5.0 for misleading potential. The proportion of ratings ≥ 4 ranged from 92% to 100% for accuracy, 90% to 99% for completeness, 98% to 100% for understandability, and 88% to 99% for misleading potential (Table 1).

The proportion of reports with high agreement, defined as all three raters independently assigning scores ≥ 4, was 91% (95% CI 83.8–95.2) for accuracy, 86% (77.9–91.5) for completeness, 98% (93.0–99.4) for understandability, and 86% (77.9–91.5) for misleading potential (Table 2).

**Figure 1:**
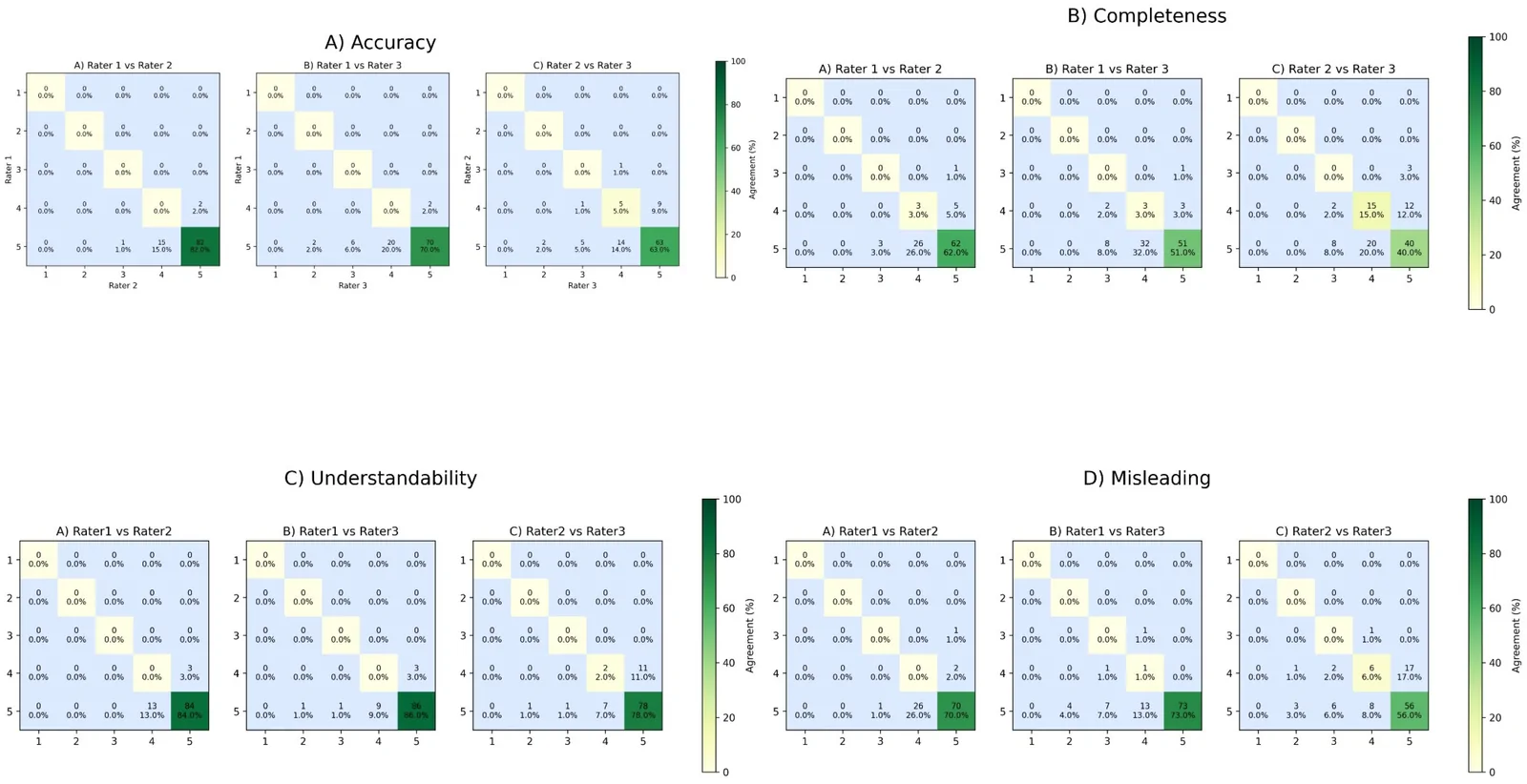
Pairwise heatmaps illustrating inter-rater exact agreement across four domains: (**A**) accuracy, (**B**) completeness, (**C**) understandability, and (**D**) misleading potential. Each panel presents pairwise comparisons between raters. Only exact agreement cells (diagonal) are color-coded from yellow to green according to agreement percentages, while non-agreement cells are displayed with a uniform light-blue background. All Likert categories (1–5), including zero-frequency cells, are shown.

Exact pairwise agreement between raters was observed almost exclusively at scores of 4 and 5, with no meaningful agreement at lower score levels. For accuracy, pairwise agreement ranged from 68% to 82% across rater pairs. Completeness showed lower agreement, ranging from 54% to 65%. In contrast, understandability demonstrated the highest agreement levels (80% to 86%), whereas pairwise agreement for misleading potential ranged from 62% to 74% (Figure 1).

Chance-corrected reliability coefficients are presented in Table 2. Gwet’s AC2 ranged from 0.931 to 0.982 across domains, whereas Krippendorff’s α ranged from −0.015 to 0.047. No report received a rating of ≤3 from more than one rater in any domain, and all ratings of 2 were assigned by the same rater (Rater 3). In the sensitivity analysis, Krippendorff’s α ranged from −0.042 to 0.047 across the nominal, ordinal, and interval distance metrics, whereas Gwet’s coefficient ranged from 0.529 to 0.826 without weighting (AC1), from 0.815 to 0.947 with linear weights, and from 0.931 to 0.982 with quadratic weights (Table S1).

**Figure 2:**
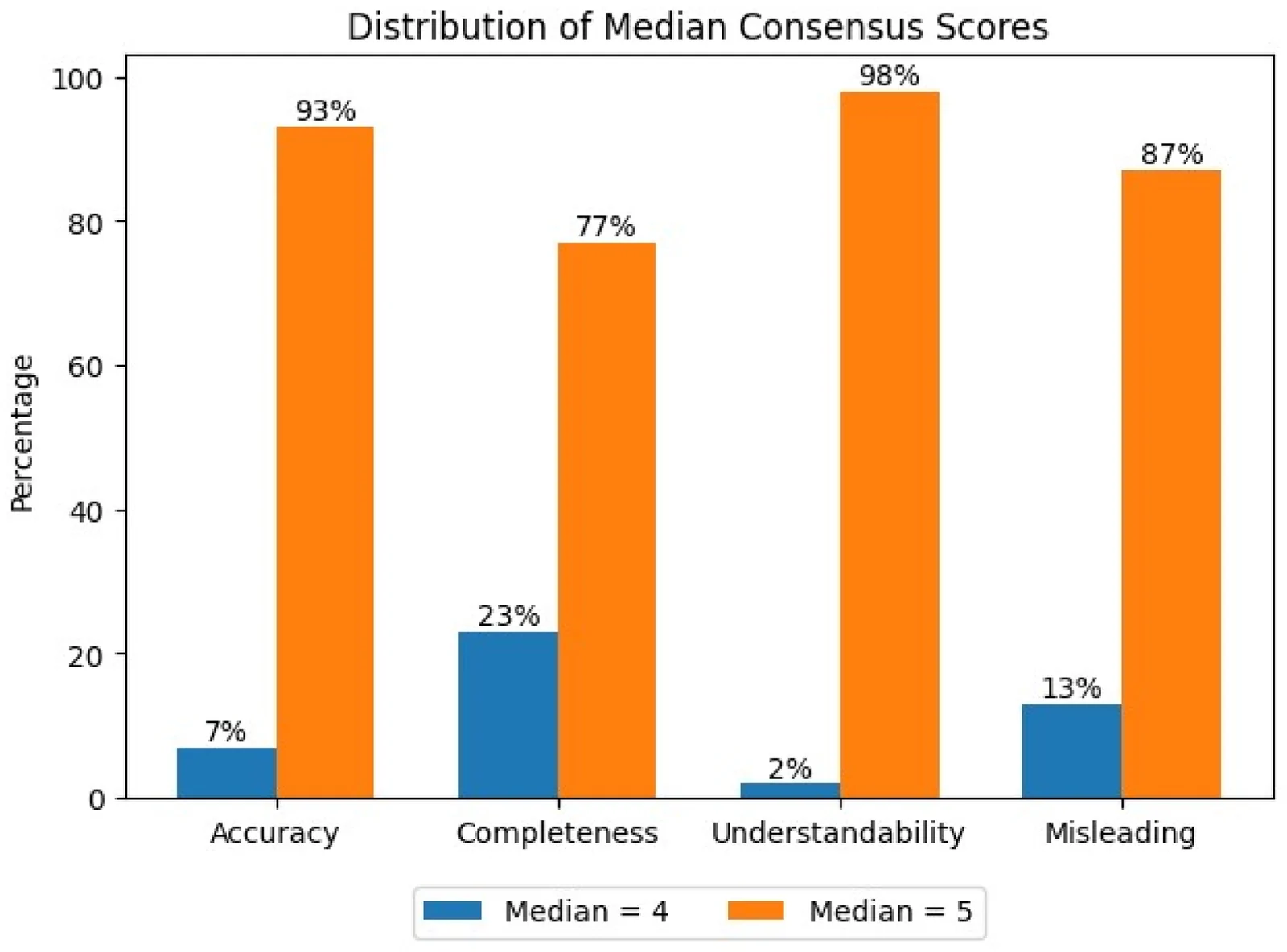
Distribution of consensus scores based on the median of three expert ratings.

When expert ratings were summarized at the report level using the median of the three raters, the majority of reports received the highest possible consensus score (5) across all domains, most notably for accuracy (93%) and understandability (98%). No report received a median consensus score below 4 in any domain; all consensus scores were either 4 or 5 (Figure 2).

At least one rating of ≤3 was assigned to 9 reports for accuracy, 14 for completeness, 2 for understandability, and 14 for misleading potential, in each case by a single rater. Ratings of 2 were given to 2 reports for accuracy, 1 for understandability, and 4 for misleading potential. On re-examination against the original reports, the deviations identified in these outputs consisted of an attenuated presentation of the original findings, either through the omission of some findings or through the understatement of their extent or severity. Of the 39 ratings of ≤3, 32 were assigned by Rater 3, and 14 of the 20 affected reports received low ratings from Rater 3 only (Table S2).

### 3.2. Volunteer Evaluation

A total of 100 non-healthcare volunteers were included in the second phase of the study. The mean age was 34.8 ± 11.9 years (median: 30.0; range: 19–70). Of the participants, 52% were female and 48% were male. Regarding educational status, 63% were university graduates and 37% were high school graduates. A history of prior shoulder MRI or shoulder surgery was reported by 20% of participants, while 80% reported no such history. When asked whether they had difficulty understanding radiology reports, 37% responded “yes,” 17% “no,” and 46% reported that they had never attempted to read such reports. Self-reported health literacy was grouped into three categories: low, moderate, and high. Low literacy (combining “very difficult” and “difficult”) was reported by 26% of participants, moderate literacy by 59%, and high literacy (combining “easy” and “very easy”) by 15%.

Cronbach’s alpha for the six questionnaire items was 0.801.

Responses to the volunteer questionnaire items are presented in Table 3. For understanding report terms, 47% of participants selected “agree” or “strongly agree” (Likert ≥ 4), including 38% and 9%, respectively. For identifying important findings, 58% of participants selected “agree” or “strongly agree” (Likert ≥ 4), including 38% and 20%, respectively. A total of 72% of participants selected “agree” or “strongly agree” for feeling informed by a doctor (43% and 29%, respectively). For understanding their shoulder condition, 56% selected “agree” or “strongly agree” (41% and 15%, respectively). Regarding understanding next steps, 75% of participants selected “agree” or “strongly agree” (42% and 33%, respectively). The highest proportion was observed for the appropriateness of the simplified text for patients, with 80% selecting “agree” or “strongly agree” (45% and 35%, respectively).

Spearman correlation analysis showed statistically significant associations between self-reported health literacy levels and the scores of the questionnaire items. Health literacy was positively correlated with the scores of understanding report terms (*r* = 0.329, *p* = 0.001), identifying important findings (*r* = 0.338, *p* = 0.001), understanding one’s condition (*r* = 0.429, *p* < 0.001), understanding next steps (*r* = 0.385, *p* < 0.001), and appropriateness of the simplified text (*r* = 0.374, *p* < 0.001). No significant correlation was observed between health literacy and the score for feeling informed by a doctor (*r* = 0.074, *p* = 0.462). Age was negatively correlated with the scores of understanding report terms (*r* = −0.352, *p* < 0.001) and understanding one’s condition (*r* = −0.306, *p* = 0.002). No significant correlations were found between age and the scores of the other questionnaire items (*p* > 0.05). After Holm adjustment, all significant correlations with health literacy (adjusted *p* ≤ 0.006) and with age (adjusted *p* ≤ 0.010) remained significant.

When scores were compared according to educational status using the Mann–Whitney U test, median scores were higher among university graduates compared to high school graduates. Statistically significant differences were observed for item 1 (*p* = 0.005), item 2 (*p* < 0.001), item 4 (*p* = 0.001), and item 6 (*p* = 0.026). No significant differences were found for item 3 (*p* = 0.369) and item 5 (*p* = 0.085). After Holm adjustment, the differences for items 1, 2, and 4 remained significant (adjusted *p* = 0.020, < 0.006, and 0.005, respectively), whereas the difference for item 6 was no longer significant (adjusted *p* = 0.078).

When scores were compared according to history of prior shoulder MRI or surgery using the Mann–Whitney U test, no statistically significant differences were observed for any of the questionnaire items (Item 1: *p* = 0.252, Item 2: *p* = 0.312, Item 3: *p* = 0.945, Item 4: *p* = 0.670, Item 5: *p* = 0.398, Item 6: *p* = 0.546).

Differences in questionnaire item scores according to self-reported difficulty in understanding radiology reports were examined using the chi-square test. No statistically significant differences were found for Item 1 (*p* = 0.899), Item 2 (*p* = 0.741), Item 3 (*p* = 0.603), Item 4 (*p* = 0.418), Item 5 (*p* = 0.195), and Item 6 (*p* = 0.093).

## 4. Discussion

The principal finding of this study is that ChatGPT-generated simplifications of shoulder MRI reports were evaluated favorably from both expert and volunteer perspectives. In the expert arm, the simplified reports were rated as accurate, complete, and understandable, with low misleading potential. In the volunteer arm, the reports were generally perceived as useful, appropriate for patients, and helpful in guiding subsequent steps. Taken together, these findings suggest that ChatGPT may be useful as an adjunctive tool for patient-facing communication of shoulder MRI reports, although the present design assessed expert ratings and perceived acceptability rather than actual patient comprehension or clinical outcomes.

From the expert perspective, these findings are relevant because patient-oriented simplification is only clinically acceptable if it preserves the diagnostic meaning of the original radiology report. In the present study, mean expert ratings for accuracy and completeness were high, and consensus median scores were 5 in the majority of reports. This suggests that the model generally succeeded in retaining clinically relevant information while rewriting the reports in simpler language. Understandability received the highest expert ratings, indicating that the outputs were not merely accurate but also linguistically accessible. At the same time, misleading potential was rated as low; however, this rating applied to each output as a whole and does not establish the clinical safety of individual statements, particularly treatment-related content.

These findings are consistent with the emerging radiology literature suggesting that LLMs can improve readability and patient-centered accessibility while preserving much of the source content. Jeblick et al. reported that radiologists generally considered ChatGPT-simplified radiology reports factually correct, largely complete, and only infrequently harmful, while also noting the possibility of omissions and misplaced emphasis [19]. More recently, Prucker et al. demonstrated in a prospective controlled trial that LLM-based simplification of oncologic CT reports improved patient comprehension and reduced reading burden, although clinically relevant errors still occurred [20]. In the musculoskeletal domain, Berzolla et al. reported that AI-simplified MRI reports improved patient comprehension and were preferred for clarity, although perceived trust was lower than for conventional reports [9]. Our findings extend this literature by focusing specifically on shoulder MRI reports and by combining structured expert review with volunteer-based evaluation in a single study.

These favorable ratings should, however, be interpreted in light of the ceiling effect and the reliability analysis. Ratings were concentrated in the highest categories, which can produce a low Krippendorff’s α despite substantial observed agreement: when nearly all ratings are 4 or 5, the agreement expected by chance is itself very high, and the few disagreements dominate kappa-type coefficients [16,17]. Gwet’s AC2 is less sensitive to this category imbalance. These different responses to the concentrated rating distribution may explain the marked discrepancy between the two coefficients [21]. Thus, the near-zero α should not be interpreted as indicating a complete absence of agreement. In the sensitivity analysis, Krippendorff’s α remained close to zero under nominal, ordinal, and interval metrics, whereas the weighting scheme substantially affected the magnitude of Gwet’s coefficient (Table S1); thus, both the chance-agreement model and the weighting scheme contributed to the divergence between the coefficients, but no distance metric brought α close to the values of Gwet’s coefficients. Nevertheless, the near-zero α indicates that the raters did not consistently agree on which reports deserved lower scores; low ratings were sparse and isolated, each being assigned by a single rater. Moreover, the assessment domains involve clinical judgment, particularly understandability and misleading potential; ratings may therefore reflect both the characteristics of the generated text and differences in raters’ expectations and interpretive thresholds. Including three independent experts allowed us to capture multiple clinical perspectives but did not eliminate rater-related variability. Thus, the high scores reflect a consistently favorable overall judgment of the outputs rather than evidence that the rating scale discriminated reliably between better and poorer simplifications. The deviations identified in low-rated outputs consisted of an attenuated presentation of the original findings, through the omission of some findings or the understatement of their extent or severity, which is consistent with the omissions and misplaced emphasis described in previous radiology LLM studies [19]. Such attenuation may be clinically relevant because it can provide false reassurance even when the remaining text is accurate. The concentration of low ratings in a single rater further suggests that raters differed in the threshold at which they penalized this type of deviation.

A further consideration is that the prompt used in this study requested not only an explanation of the findings but also general information on treatment and on what the patient should do or avoid. This content goes beyond linguistic simplification, because management advice is not usually contained in the radiology report and depends on clinical information, such as symptoms, examination findings, age, and activity level, that was not available to the model. Although this content was included in the overall expert assessment, the treatment-related statements were not evaluated separately for factual support and clinical safety. AI-based chatbots can provide general information and exercise recommendations for patients with various chronic conditions; however, such recommendations have shown gaps in comprehensiveness, accuracy, and readability [22]. Treatment-related content generated by LLMs should therefore be regarded as general information only and should not guide individual treatment decisions without physician evaluation. Nevertheless, these questions reflect what the public wants to know after reading an imaging report, and describing how an LLM answers them is relevant because patients are likely to ask such questions regardless of whether a clinically appropriate answer can be given without examination.

However, not all aspects of volunteer perception were equally strong. Compared with patient appropriateness and guidance regarding next steps, lower scores were observed for understanding of medical terminology and for full comprehension of the underlying shoulder condition. This suggests that simplifying a radiology report may improve the perceived reading experience without necessarily ensuring full understanding of pathology-specific concepts; moreover, because the questionnaire measured self-reported understanding, these findings reflect perceived rather than objective comprehension. Shoulder MRI reports often involve nuanced distinctions among tendinopathy, partial tearing, labral abnormalities, bursitis, impingement-related findings, and degenerative changes. Even when these are rewritten in simplified language, some degree of conceptual complexity is likely to remain. Thus, LLM-generated simplification should be considered a supportive communication layer rather than a replacement for physician explanation. It should be emphasized that AI-generated texts cannot replace a face-to-face consultation, in which the physician integrates the imaging findings with the patient’s history, symptoms, and physical examination.

The subgroup analyses also deserve attention. Participants who reported a better ability to understand health-related information had higher scores on most questionnaire items, which is consistent with the broader literature on health literacy and medical communication. Conversely, older age was associated with slightly lower scores for understanding report terminology and one’s own condition. Although these effects were modest, they suggest that individual factors such as age and baseline health literacy may influence how simplified AI-generated reports are interpreted, and that such outputs may not be understood uniformly across all user groups. This underlines the need for digital health information, including AI-generated explanations, to be adapted to readers with different ages, educational backgrounds, and health literacy levels. Of note, no statistically significant differences were observed in questionnaire scores according to self-reported difficulty in understanding radiology reports, suggesting that the simplified content may be perceived similarly across individuals with different baseline comprehension levels. This raises the possibility that AI-assisted text simplification may produce a relatively homogeneous reading experience across a broad lay audience. However, these subgroup analyses were exploratory, and the difference according to education for item 6 did not remain significant after correction for multiple testing.

Although AI-based tools are increasingly used, conventional online sources such as YouTube and health-related websites remain among the most popular sources of health information, and their content is often of variable quality and reliability [4,5]. LLM-based chatbots add a further risk: they can generate fluent and confident but inaccurate statements, so-called hallucinations, which lay readers may find difficult to recognize [23,24]. When such outputs concern medical findings or treatment, they may lead to delayed care, inappropriate self-management, or unnecessary anxiety, with potentially irreversible consequences for individual and public health. Moreover, unlike a general video or website, an LLM-generated explanation of a patient’s own report appears personalized, which may further increase the trust placed in it. These risks reinforce the need for clinician oversight and clear disclosure whenever LLM-generated simplifications are offered to patients.

This study has several strengths. First, it focused specifically on shoulder MRI, a common and clinically relevant imaging modality in orthopedic practice. Second, it used 100 real reports from routine clinical care rather than a small convenience sample. Third, it incorporated two complementary perspectives: structured expert review and volunteer-based layperson evaluation. This dual approach allowed us to examine not only whether the reports were perceived as understandable by lay readers but also whether they remained clinically acceptable and potentially useful.

### 4.1. Limitations

This study has several limitations. First, this was a single-center retrospective text-evaluation study of 100 reports obtained with a single 1.5-T scanner, which may limit generalizability to other institutions, scanners, reporting styles, subspecialties, or languages; in addition, the distribution of individual MRI findings was not systematically extracted. Second, the simplified outputs were generated using a single model configuration and a single standardized prompt; results may differ with other models or prompting strategies. Moreover, each report was processed once with a web-based model using default settings that may be updated over time; repeated-output reproducibility was therefore not assessed, and the results may not be fully reproducible with later model versions. Third, volunteers evaluated only the simplified reports and did not directly compare them with the original radiology reports; therefore, the present study addresses perceived utility rather than comparative superiority over standard reporting. Furthermore, the volunteers were not patients evaluating their own reports, the questionnaire was study-specific and not formally validated, and it measured self-reported rather than objective comprehension; therefore, the study cannot demonstrate that ChatGPT improves patient comprehension. In addition, because each volunteer evaluated a different report, the volunteer ratings may partly reflect differences in the complexity and content of the individual reports, such as the number and severity of findings, rather than characteristics of the volunteers alone; report complexity was not quantified, and the design did not allow variability between reports to be separated from variability between volunteers. Fourth, although expert-rated misleading potential was low, the study did not assess downstream behavioral or emotional consequences in real clinical settings. The prompt also requested general treatment-related guidance, which was not evaluated separately for factual support and clinical safety, because the appropriateness of such recommendations cannot be adjudicated without clinical information that was not available in this text-based design. Fifth, although expert evaluation was favorable, simplification errors remain possible, as highlighted in prior radiology LLM studies [23,24]. Sixth, the expert panel consisted of orthopedic surgeons and did not include musculoskeletal radiologists, who may judge the preservation of radiological meaning differently. Seventh, the original radiology reports were used as the reference standard without independent re-review of the MRI images; therefore, the study assessed fidelity to the source report rather than diagnostic accuracy, and any errors in the original reports would not have been detected. Finally, the marked ceiling effect limited the ability of the rating scale to discriminate between outputs, as reflected by the near-zero Krippendorff’s α despite high observed agreement; the expert ratings should therefore be interpreted as a consistently favorable overall judgment rather than as evidence of reliable discrimination between outputs.

### 4.2. Clinical Implications and Future Directions

Despite these limitations, the present findings have practical implications. As patient access to radiology reports continues to expand through digital systems and patient portals, there is increasing demand for communication tools that bridge the gap between specialist language and patient understanding. Our results suggest that ChatGPT-based simplification may help meet this need, particularly as a supplementary explanation tool accompanying the formal radiology report. A plausible future workflow would involve an AI-generated patient-friendly explanation presented alongside the original report, ideally with clinician oversight and clear disclosure that the simplified text is supportive rather than definitive. Such an approach may improve patient engagement, reduce confusion, and facilitate more structured clinical conversations.

Future research should build on these findings in several directions. Prospective comparative studies should evaluate whether LLM-generated simplifications improve actual patient understanding, recall, satisfaction, anxiety, or decisional confidence compared with standard reports alone. Multicenter studies would clarify generalizability across institutions and reporting cultures. Additional work is also needed to determine how best to optimize prompting strategies, how to handle uncertainty and incidental findings, and whether pathology-specific simplification approaches may further improve comprehension. Studies involving symptomatic shoulder patients would also provide a more clinically relevant picture of real-world performance. Future studies should also include musculoskeletal radiologists in the expert panel, use validated or knowledge-based comprehension measures, assess the reproducibility of outputs across repeated runs and model versions, and evaluate treatment-related content separately for factual support and clinical safety. Assigning each report to several volunteers and quantifying report complexity would also allow report-level and reader-level effects to be distinguished.

## 5. Conclusions

ChatGPT-generated simplifications of shoulder MRI reports were rated favorably by senior orthopedic surgeons for accuracy, completeness, understandability, and misleading potential. Non-healthcare volunteers likewise perceived the simplified reports as useful, appropriate for patients, and helpful in understanding next steps, although self-reported understanding of medical terminology and of the underlying shoulder pathology was less favorable than the other items. However, these findings reflect expert ratings and perceived acceptability; they do not demonstrate improved patient comprehension or clinical safety. Because the model had no access to the patient’s history, symptoms, or examination findings, LLM-generated explanations cannot provide individualized risk–benefit assessment or management recommendations. LLM-generated simplifications are therefore unsuitable for independent use or as a primary source of information in clinical decision-making, and may at most serve as a supplementary tool in patient-centered radiology communication. Such outputs should nonetheless be regarded as supportive rather than definitive, and clinician oversight remains essential to ensure safe and accurate patient-facing use.

## Figures and Tables

**Table 1:** Expert assessment of the quality of ChatGPT-generated simplified reports (*N* = 100 MRI reports). For all domains, higher scores indicate more favorable ratings (for misleading potential, 5 = very low risk).

	Rater 1			Rater 2			Rater 3		
	Mean ± SD	Median (Min–Max)	Likert ≥ 4, *n* (%)	Mean ± SD	Median (Min–Max)	Likert ≥ 4, *n* (%)	Mean ± SD	Median (Min–Max)	Likert ≥ 4, *n* (%)
Accuracy	5.0 ± 0.1	5.0 (4.0–5.0)	100 (100.0)	4.8 ± 0.4	5.0 (3.0–5.0)	99 (99.0)	4.6 ± 0.7	5.0 (2.0–5.0)	92 (92.0)
Completeness	4.9 ± 0.3	5.0 (3.0–5.0)	99 (99.0)	4.7 ± 0.5	5.0 (3.0–5.0)	97 (97.0)	4.5 ± 0.7	5.0 (3.0–5.0)	90 (90.0)
Understandability	5.0 ± 0.2	5.0 (4.0–5.0)	100 (100.0)	4.9 ± 0.3	5.0 (4.0–5.0)	100 (100.0)	4.9 ± 0.4	5.0 (2.0–5.0)	98 (98.0)
Misleading potential	5.0 ± 0.2	5.0 (3.0–5.0)	99 (99.0)	4.7 ± 0.5	5.0 (3.0–5.0)	99 (99.0)	4.6 ± 0.8	5.0 (2.0–5.0)	88 (88.0)

**Table 2:** Inter-rater agreement and reliability across evaluation domains.

Domain	All Three Raters ≥ 4, *n* (%; 95% CI)	Exact Pairwise Agreement, Range (%)	Krippendorff’s α, Ordinal (95% CI)	Gwet’s AC2 (95% CI)
Accuracy	91 (91.0; 83.8–95.2)	68–82	−0.001 (−0.098 to 0.098)	0.959 (0.938–0.975)
Completeness	86 (86.0; 77.9–91.5)	54–65	−0.010 (−0.105 to 0.085)	0.931 (0.909–0.950)
Understandability	98 (98.0; 93.0–99.4)	80–86	−0.015 (−0.097 to 0.084)	0.982 (0.970–0.991)
Misleading potential	86 (86.0; 77.9–91.5)	62–74	0.047 (−0.047 to 0.133)	0.941 (0.913–0.963)

CI, confidence interval. Exact pairwise agreement is shown as the range across the three rater pairs. The 95% CIs for Krippendorff’s α and Gwet’s AC2 were estimated by bootstrapping (5000 resamples of reports). Coefficients calculated with alternative distance metrics and weighting schemes are provided in Table S1.

**Table 3:** Responses of non-healthcare volunteers to the questionnaire items (*N* = 100).

Item	Scores					Likert ≥ 4, *n* (%; 95% CI)
	1, *n* (%)	2, *n* (%)	3, *n* (%)	4, *n* (%)	5, *n* (%)	
**Item 1: I was able to understand the terms in the report.**	6 (6.0)	8 (8.0)	39 (39.0)	38 (38.0)	9 (9.0)	47 (47.0; 37.5–56.7)
Item 2: I clearly understood which findings were important or unimportant.	3 (3.0)	9 (9.0)	30 (30.0)	38 (38.0)	20 (20.0)	58 (58.0; 48.2–67.2)
Item 3: I felt as if I was informed by a doctor.	0 (0.0)	6 (6.0)	22 (22.0)	43 (43.0)	29 (29.0)	72 (72.0; 62.5–79.9)
Item 4: I clearly understood my shoulder condition/problem.	2 (2.0)	15 (15.0)	27 (27.0)	41 (41.0)	15 (15.0)	56 (56.0; 46.2–65.3)
Item 5: It helped me understand what I should do next.	0 (0.0)	3 (3.0)	22 (22.0)	42 (42.0)	33 (33.0)	75 (75.0; 65.7–82.5)
Item 6: I found the simplified text appropriate for patients.	0 (0.0)	3 (3.0)	17 (17.0)	45 (45.0)	35 (35.0)	80 (80.0; 71.1–86.7)

Responses were rated on a 5-point Likert scale (1 = strongly disagree, 2 = disagree, 3 = neutral, 4 = agree, 5 = strongly agree). Likert ≥ 4 indicates agreement (agree or strongly agree). CI, confidence interval (Wilson score method).

## Data Availability

The anonymized individual expert ratings are provided in Data S1. The de-identified original reports and the ChatGPT-generated outputs are available on request from the corresponding author.

## Supplementary Materials

The following supporting information can be downloaded at: https://www.mdpi.com/article/10.3390/diagnostics16213609/s1, Figure S1: Flow diagram of report selection and evaluation; Table S1: Inter-rater reliability coefficients calculated with alternative distance metrics and weighting schemes; Table S2: Reports that received at least one rating of ≤3, with the corresponding domain, score, and rater; Data S1: Anonymized individual expert ratings.

## Abbreviations

The following abbreviations are used in this manuscript:

MRI	Magnetic resonance imaging
LLM	Large language model
CI	Confidence interval
