# Supplementary material for: Evaluation of ChatGPT-Generated Simplifications of Shoulder MRI Reports: Assessments by Orthopedic Surgeons and Non-Healthcare Volunteers
Source: Diagnostics (Basel). 2026 Nov 1;16(21):3609. doi: 10.3390/diagnostics16213609 (PMC13762333; doi:10.3390/diagnostics16213609)
Supplement: Supplementary file 1 [file diagnostics-16-03609-s001.zip › diagnostics-4581826_Supplementary_Materials_1.pdf]

Supplementary Materials

Evaluation of ChatGPT-Generated Simplifications of Shoulder MRI Reports: Assessments by Orthopedic Surgeons and Non-Healthcare Volunteers

Figure S1. Flow diagram of report selection and evaluation.

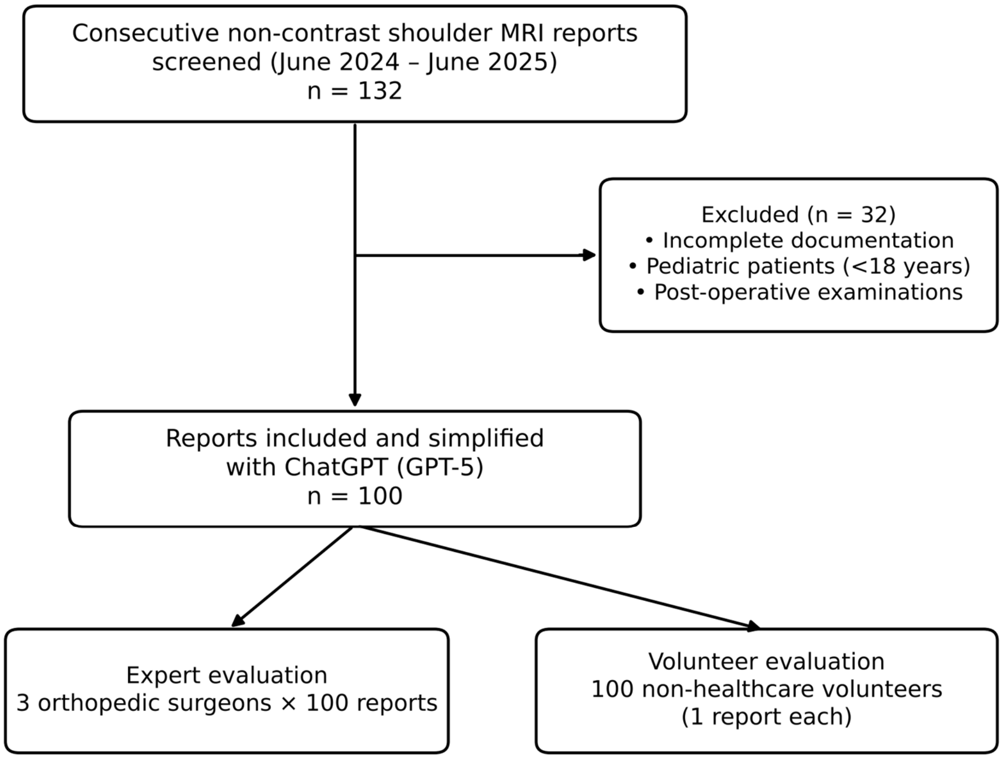

Table S1. Inter-rater reliability of the expert ratings calculated with alternative distance metrics and weighting schemes (three raters, 100 reports). Values are estimates with bootstrap 95% confidence intervals (5000 resamples of reports).

Coefficient	Accuracy	Completeness	Understandability	Misleading potential
Krippendorff α (nominal)	0.006 (−0.075 to 0.088)	0.030 (−0.055 to 0.116)	−0.007 (−0.088 to 0.093)	0.030 (−0.044 to 0.103)
Krippendorff α (ordinal)	−0.001 (−0.098 to 0.098)	−0.010 (−0.105 to 0.085)	−0.015 (−0.097 to 0.084)	0.047 (−0.047 to 0.133)
Krippendorff α (interval)	−0.022 (−0.095 to 0.071)	−0.042 (−0.128 to 0.048)	−0.025 (−0.085 to 0.062)	0.016 (−0.063 to 0.099)
Fleiss' κ	0.003 (−0.079 to 0.085)	0.026 (−0.059 to 0.113)	−0.011 (−0.092 to 0.090)	0.027 (−0.047 to 0.100)
Gwet AC1 (unweighted)	0.714 (0.637 to 0.785)	0.529 (0.449 to 0.609)	0.826 (0.767 to 0.883)	0.659 (0.574 to 0.740)
Gwet AC2 (linear)	0.896 (0.859 to 0.927)	0.815 (0.772 to 0.855)	0.947 (0.924 to 0.967)	0.864 (0.817 to 0.903)
Gwet AC2 (quadratic)	0.959 (0.938 to 0.975)	0.931 (0.909 to 0.950)	0.982 (0.970 to 0.991)	0.941 (0.913 to 0.963)
Reports with identical ratings from all three raters, n (%)	61 (61.0)	39 (39.0)	75 (75.0)	56 (56.0)

Krippendorff's α and Fleiss' κ estimate chance agreement from the pooled distribution of ratings; Gwet's coefficients use a different chance-agreement model that is less sensitive to concentration of ratings in a few categories. The ordinal Krippendorff's α and the quadratic-weighted Gwet's AC2 correspond to the coefficients reported in Table 2 of the main text.

Table S2. Reports that received at least one rating of ≤ 3 from any rater (20 of 100 reports), with the domain, score, and rater.

Report	Ratings ≤ 3: domain: score (rater)
2	Completeness: 3 (Rater 3)
5	Accuracy: 3 (Rater 3); Completeness: 3 (Rater 3); Misleading potential: 3 (Rater 3)
7	Accuracy: 2 (Rater 3); Completeness: 3 (Rater 3); Misleading potential: 2 (Rater 3)
8	Misleading potential: 3 (Rater 3)
12	Accuracy: 2 (Rater 3); Completeness: 3 (Rater 3); Misleading potential: 2 (Rater 3)
14	Completeness: 3 (Rater 3)
15	Accuracy: 3 (Rater 3); Completeness: 3 (Rater 3); Misleading potential: 2 (Rater 3)
18	Completeness: 3 (Rater 3); Understandability: 3 (Rater 3); Misleading potential: 3 (Rater 3)
21	Completeness: 3 (Rater 3); Misleading potential: 3 (Rater 3)
26	Accuracy: 3 (Rater 3)
32	Completeness: 3 (Rater 1)
43	Misleading potential: 3 (Rater 3)
46	Misleading potential: 3 (Rater 3)
48	Accuracy: 3 (Rater 3); Completeness: 3 (Rater 2); Misleading potential: 3 (Rater 3)
51	Accuracy: 3 (Rater 3); Completeness: 3 (Rater 3); Understandability: 2 (Rater 3); Misleading potential: 2 (Rater 3)
62	Accuracy: 3 (Rater 3); Completeness: 3 (Rater 3); Misleading potential: 3 (Rater 3)
69	Accuracy: 3 (Rater 2); Misleading potential: 3 (Rater 2)
80	Misleading potential: 3 (Rater 1)
83	Completeness: 3 (Rater 2)
89	Completeness: 3 (Rater 2)

Scores are on a 5-point Likert scale; for misleading potential, lower scores indicate higher risk. No report received a rating of ≤ 3 from more than one rater in the same domain. On re-examination against the original reports, the deviations identified in these outputs consisted of an attenuated presentation of the original findings, through the omission of some findings or the understatement of their extent or severity.

Number of ratings ≤ 3 by domain (Rater 1/Rater 2/Rater 3): Accuracy, 0/1/8; Completeness, 1/3/10; Understandability, 0/0/2; Misleading potential, 1/1/12. Total: 2/5/32 (39 ratings in 20 reports).
